# Supplementary material for: Leveraging Zebrafish Embryo Phenotypic Observations to Advance Data-Driven Analyses in Toxicology
Source: Environ Sci Technol. 2025 Feb 27;59(9):4304–17. doi: 10.1021/acs.est.4c11757 (PMC11912306; doi:10.1021/acs.est.4c11757)
Supplement: Supplementary file 1 — es4c11757_si_001.pdf [file es4c11757_si_001.pdf]

Supporting Information for:

Leveraging zebrafish embryo phenotypic observations to advance data-driven analyses in toxicology

Authors: Paul Michaelis[1,#], Nils Klüver[1,#,*], Silke Aulhorn[1], Hannes Bohring[2,3], Jan Bumberger[2,4], Kristina Haase[2,3], Tobias Kuhnert[2,3], Eberhard Küster[1], Janet Krüger[1], Till Luckenbach[1], Riccardo Massei[1,2,4], Lukas Nerlich[1], Sven Petruschke[2,3], Thomas Schnicke[2,3], Anton Schnurpel[1], Stefan Scholz[1], Nicole Schweiger[1], Daniel Sielaff[2,3], Wibke Busch[1#]

[1] Helmholtz Centre for Environmental Research - UFZ, Department Ecotoxicology, Permoserstraße 15, 04318 Leipzig, Germany

[2] Helmholtz Centre for Environmental Research - UFZ, Research Data Management - RDM, Permoserstraße 15, 04318 Leipzig, Germany

[3] Helmholtz Centre for Environmental Research - UFZ, IT Department, Permoserstraße 15, 04318 Leipzig, Germany

[4] Helmholtz Centre for Environmental Research - UFZ, Department Monitoring and Exploration Technologies, Permoserstraße 15, 04318 Leipzig, Germany

[#] equal contribution

[*] corresponding authors: nils.kluever@ufz.de, wibke.busch@ufz.de

Contents:

Tables S1 to S3
Figures S1 to S6
References

Table S1: Experimental metadata information in INTOB.

Metadata information	Variable #1	Variable #2	Variable #3	Variable #4
Substance	Name	DTXSID	Molecular Mass	-
Test concentrations	Number of concentrations	Test concentrations	Unit	-
Fish strain	Name of fish strain	-	-	-
Exposure volume	Volume per embryo in ml	-	-	-
Exposure temperature	Temperature in °C	-	-	-
Medium	Name	-	-	-
pH in medium at start	pH Value	-	-	-
Buffer	Name of buffer	Concentration in mmol/L	-	-
Solvent	Yes/No	Name	DTXSID	proportion in test % (v/v)
Experimenter	Name of experimenter	-	-	-
Exposure start	Age at exposure start in hpf	-	-	-
Additional information	Free text information	-	-	-
Plate configuration	Type (well plate format; Exposure vial)	Number of well plates/Vials	If vial, number of embryos per vial	-

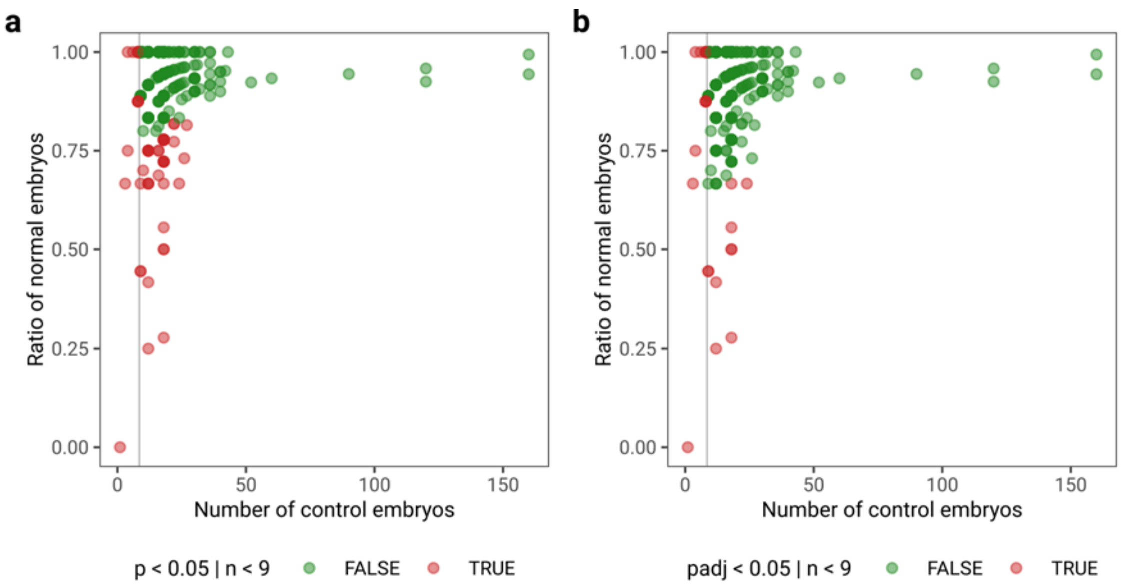

Figure S1: Assessment of embryo quality with and without p-value adjustment. Green and red dots represent experiments that are identified as high- or low-quality experiments, respectively. The vertical line represents a cutoff for low sample size at 9 embryos. **a**, experiments with p-values < 0.05 in binomial tests or fewer than 9 embryos were identified as low-quality experiments. **b**, with p-value adjustment (Benjamini & Hochberg), the tests are more stringent in identifying low-quality experiments. In turn, they are potentially more lenient towards false negatives, i.e. low-quality experiments that are not identified as such.

Table S2: Effect vocabulary used in the literature review (Data_file_S1_zotero_phenotype_library).

**Observation**	**Abbreviation**	**Keywords**
Abnormal Behavior	AB	behavior, (tail) contraction, movement, shivering, tremor, immobility, shoaling, scototaxis, thigmotaxis, sensitivity, swimming, activity, photomotor, locomotor, response, velocity
Abnormal Blood Circulation	AC	blood, circulation, congestion, flow, pooling, hemoglobin
Abnormal Eye	AE	eye, ocular, retina, optic
Abnormal Hatching	AH	hatching
Abnormal Intestine	AI	intestine, gut
Abnormal Pigmentation	AP	pigmentation, chromatophore
Abnormal Swim Bladder	AS	swim bladder, inflation
Abnormal Tail Effects	AT	tail, detachment, somites, fin, dorsal, caudal, pectoral, pelvic, anal, tip
Coagulation	CO	coagulation, apoptosis, necrosis
Developmental Delay	DD	development, retardation
Edema	ED	edema, pericardial, yolk (sac)
Gene Regulation	GR	gene, expression, regulation
Heart	HE	heart, beat, cardiac
Malformation	MA	head, scoliosis, lordosis, kyphosis, chorda, sacculi/otoliths, octavolateral, snout, body, axis, jaw, spine, arches, brain, cartilage, skeletal, liver, hepar, haemorrhage, facial, trabecular, palate, epiboly, trunk, pancreas, endoderm, kidney
Mortality Rate	MR	mortality, survival, LC, NOEC, LOEC, NOAEL, LEL, LEC
Neuronal Defects	ND	neurons, neurotransmitter, memory, cognitive

Table S3: Columns used in the Zotero library to store information (Data_file_S1_zotero_phenotype_library).

Zotero column name	Value
Call Number	Name of substance tested (IUPAC or common abbreviation), substances in mixtures are separated with semicolons
Archive	Observed effects, comma separated
Location in Archive	Time points at which observations were made in hours post fertilization (hpf), comma separated
Series Title	Fish strain, assumed WT if not otherwise specified

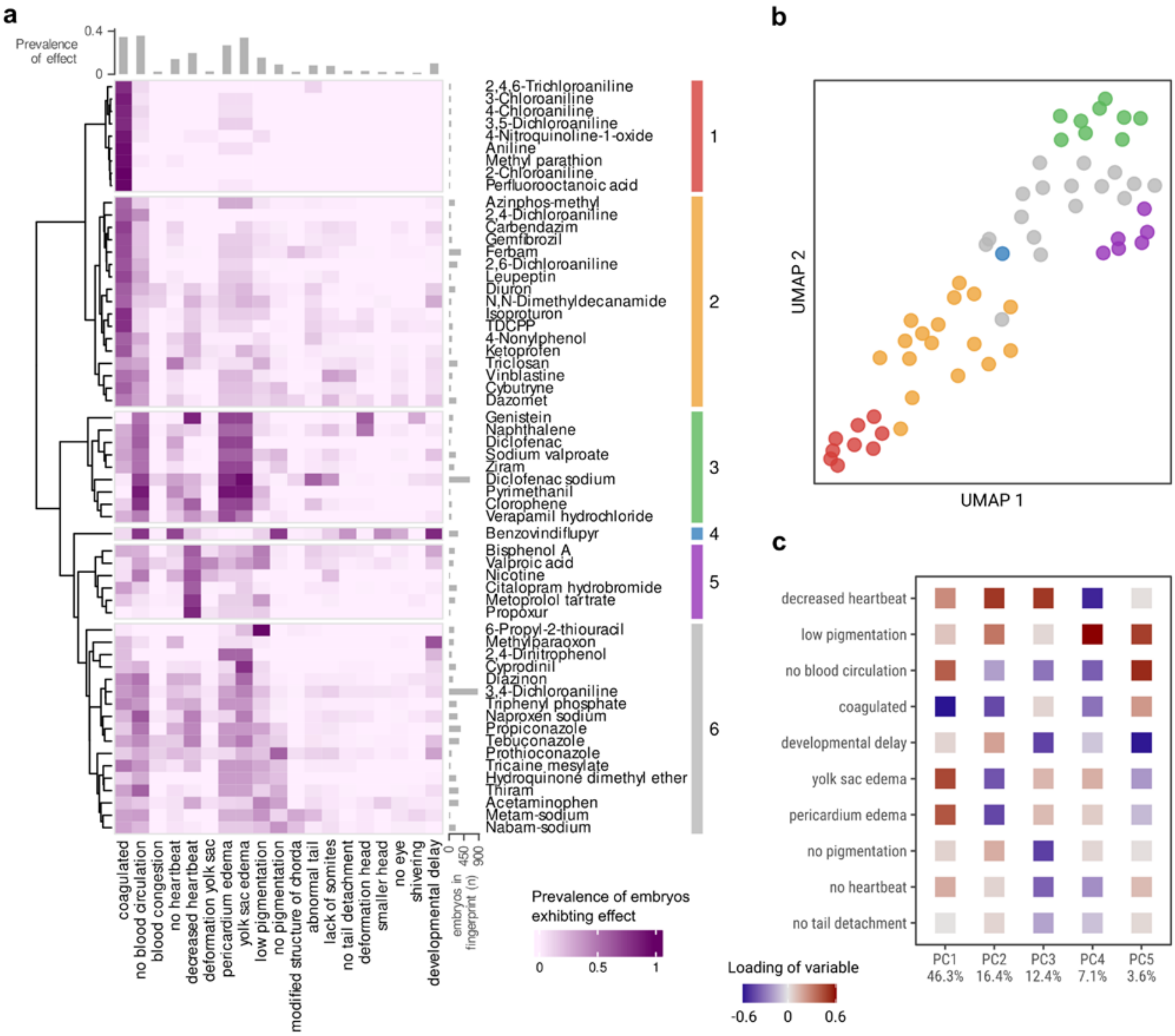

Figure S2: Phenotypic fingerprinting of chemical effects at 48 hpf. **a**, clustered heatmap of phenotypic fingerprints with values representing the proportion of embryos exhibiting an effect when exposed to concentrations between the EC5 and LC99 of the respective substance. Some substance names were shortened for brevity: n-(1,3-Dimethylbutyl)-N'-phenyl-p-phenylenediamine (6-PPDQ), ethyl 3-aminobenzoate methanesulfonic acid salt (tricaine mesylate), and tris(1,3-dichloro-2-propyl) phosphate (TDCPP). **b**, UMAP embedding of the fingerprints for visualisation of relations between clusters. **c**, the 10 most important effects for explaining variance based on the Euclidean norm of the first five principal components.

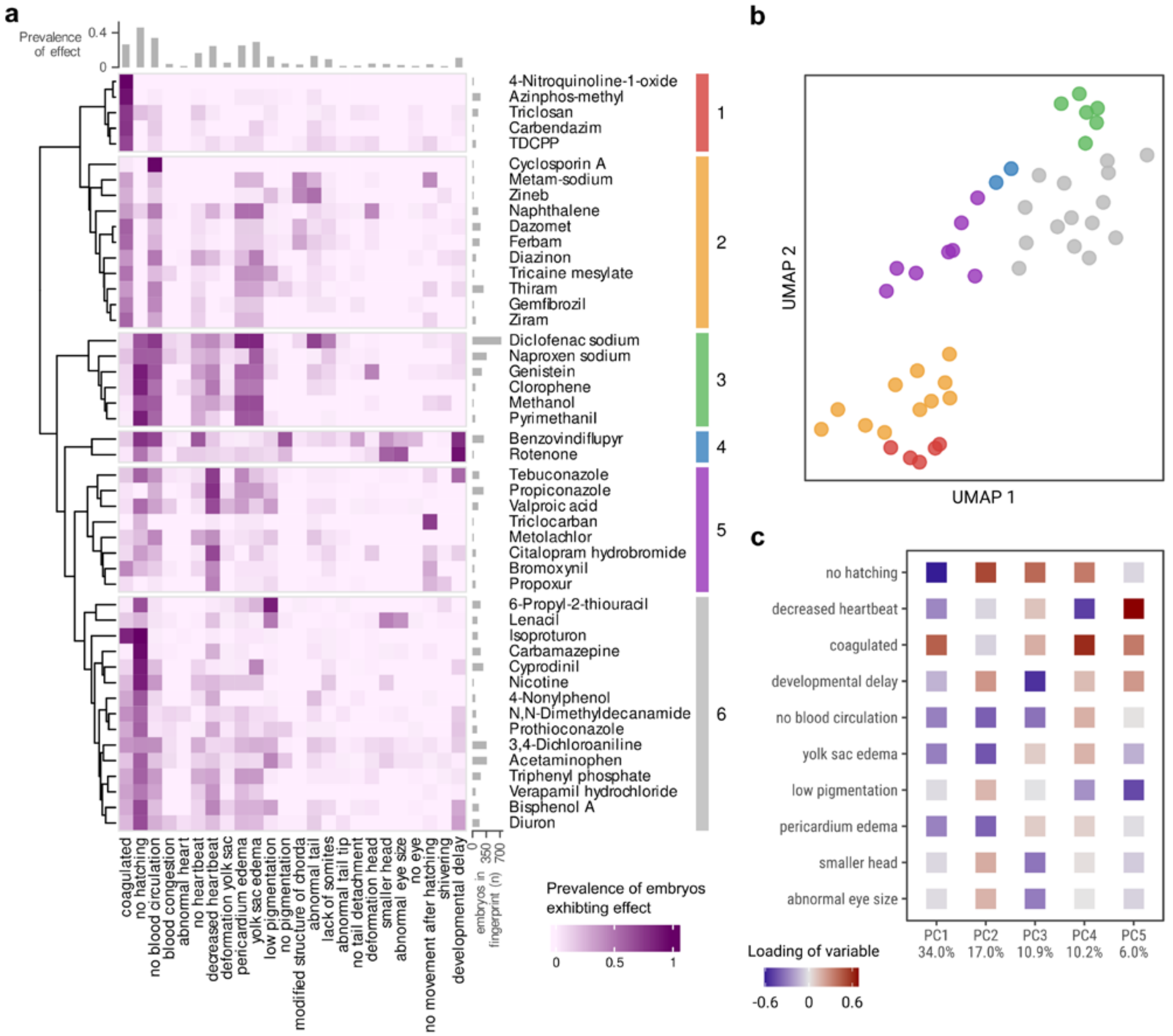

Figure S3: Phenotypic fingerprinting of chemical effects at 72 hpf. **a**, clustered heatmap of phenotypic fingerprints with values representing the proportion of embryos exhibiting an effect when exposed to concentrations between the EC5 and LC99 of the respective substance. Some substance names were shortened for brevity: n-(1,3-Dimethylbutyl)-N'-phenyl-p-phenylenediamine (6-PPDQ), ethyl 3-aminobenzoate methanesulfonic acid salt (tricaine mesylate), and tris(1,3-dichloro-2-propyl) phosphate (TDCPP). **b**, UMAP embedding of the fingerprints for visualisation of relations between clusters. **c**, the 10 most important effects for explaining variance based on the Euclidean norm of the first five principal components.

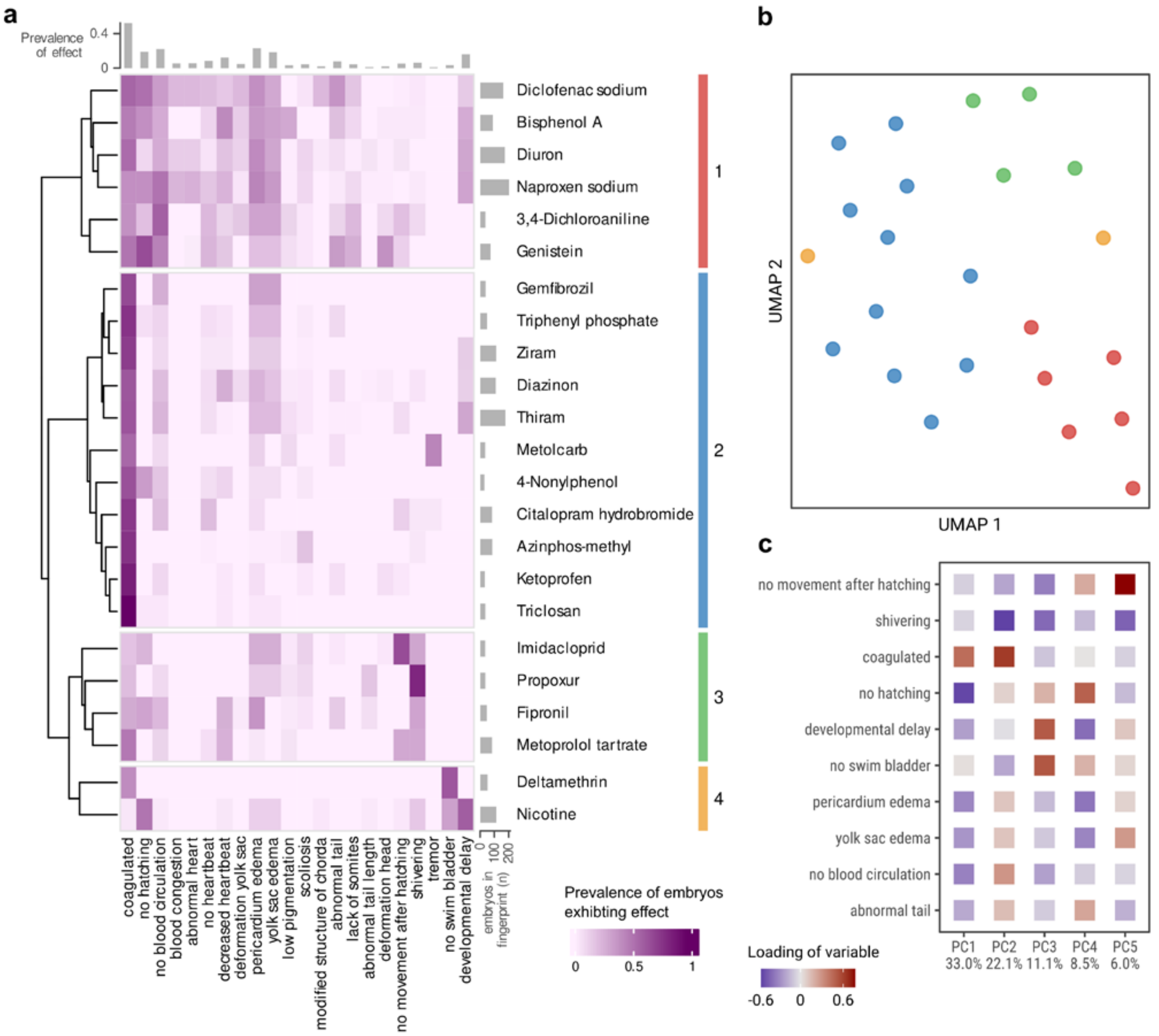

Figure S4: Phenotypic fingerprinting of chemical effects at 120 hpf. **a**, clustered heatmap of phenotypic fingerprints with values representing the proportion of embryos exhibiting an effect when exposed to concentrations between the EC5 and LC99 of the respective substance. Some substance names were shortened for brevity: n-(1,3-Dimethylbutyl)-N'-phenyl-p-phenylenediamine (6-PPDQ), ethyl 3-aminobenzoate methanesulfonic acid salt (tricaine mesylate), and tris(1,3-dichloro-2-propyl) phosphate (TDCPP). **b**, UMAP embedding of the fingerprints for visualisation of relations between clusters. **c**, the 10 most important effects for explaining variance based on the Euclidean norm of the first five principal components.

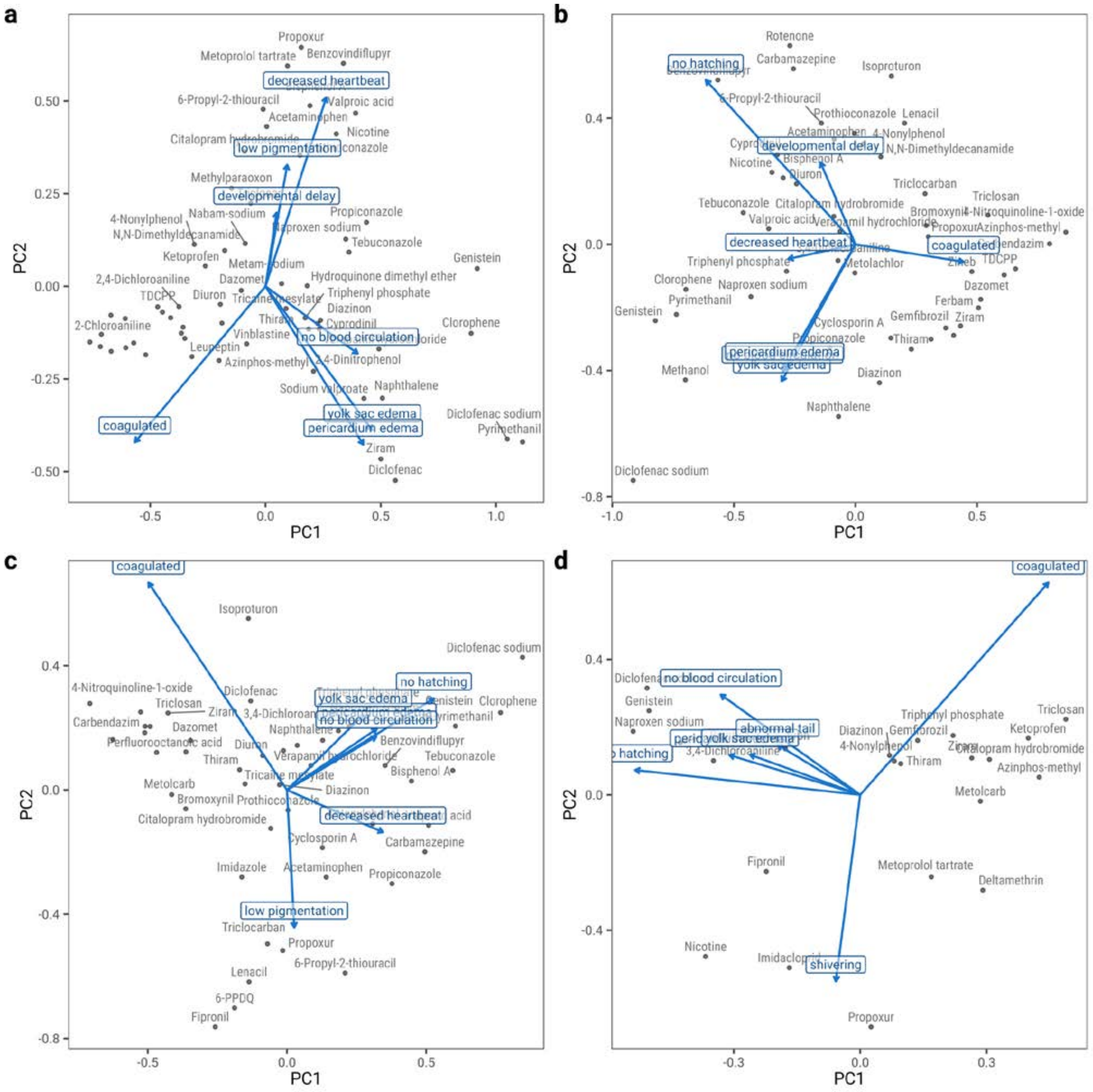

Figure S5: PCAs of phenotypic fingerprints. Magnitude and direction of the loadings in the first two principal components for the 7 most important variables are shown in blue. Some points are not labeled due to too many overlaps. Some substance names were abbreviated or changed to shorter synonyms: N-(1,3-Dimethylbutyl)-N'-phenyl-p-phenylenediamine (6-PPDQ), ethyl 3-aminobenzoate methanesulfonic acid salt (tricaine mesylate) and tris(1,3-dichloro-2-propyl) phosphate (TDCPP). **a**, **b**, **c** and **d** show PCAs for 48, 72, 96 and 120 hpf, respectively.

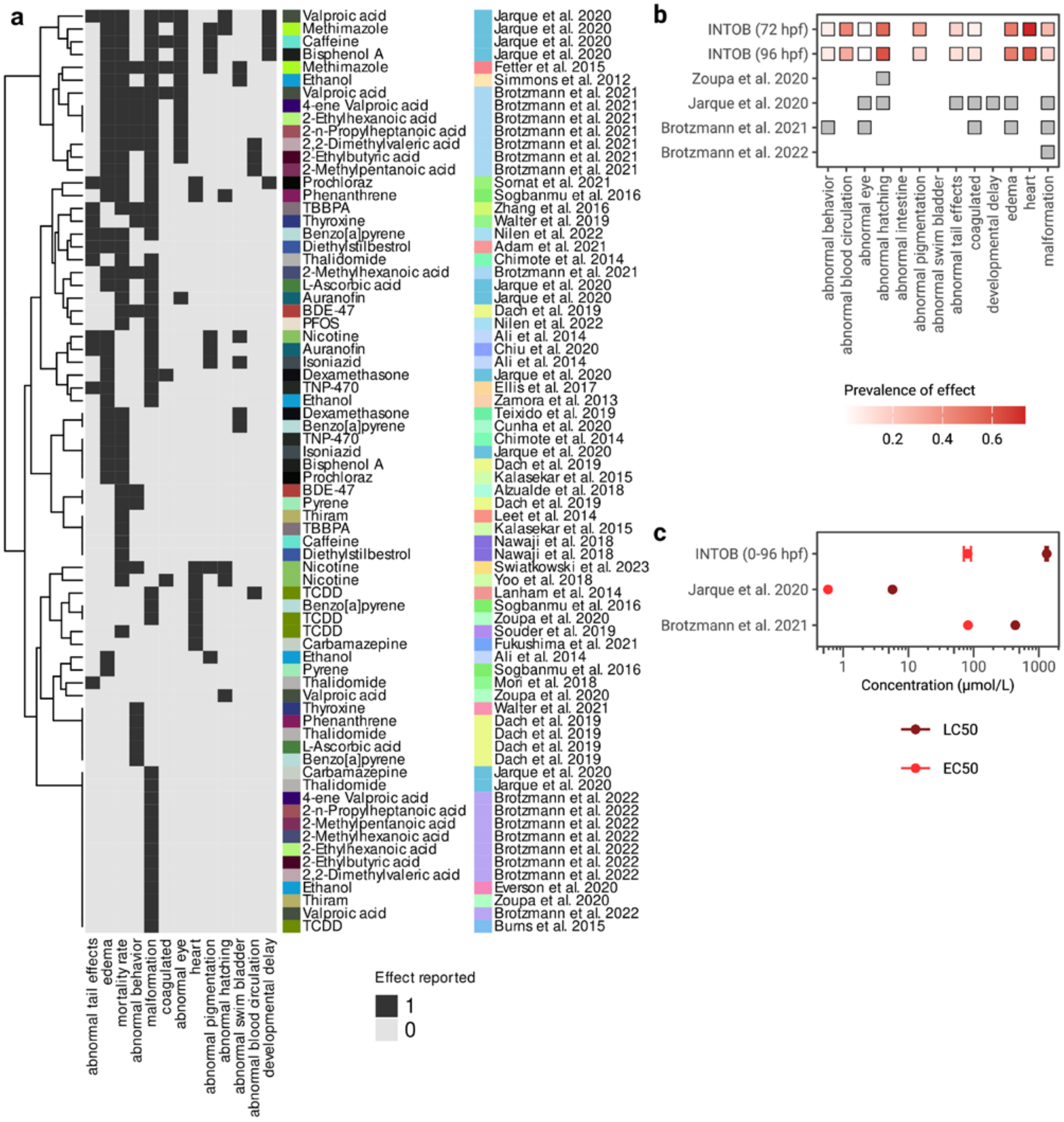

Figure S6: Comparison of effect patterns from literature. **a**, substances assessed in more than one study and with observations at 72, 96 and/or 120 hpf are compared and clustered by hierarchical clustering[1–30]. Abbreviations: tetrabromobisphenol A (TBBPA), 2,2',4,4'-tetrabromodiphenyl ether (BDE-47), O-(chloroacetylcarbamoyl)fumagillol (TNP-470), perfluorooctanesulfonic acid (PFOS), 2,3,7,8-tetrachlorodibenzo-P-dioxin (TCDD). **b**, phenotypic fingerprints from INTOB observational data and effect patterns from literature for valproic acid. **c**, EC50 and LC50 values for valproic acid modeled from INTOB observational data and reported in literature.

References

(1) Adam, A. H. B.; de Haan, L. H. J.; Louisse, J.; Rietjens, I.; Kamelia, L. Assessment of the in Vitro Developmental Toxicity of Diethylstilbestrol and Estradiol in the Zebrafish Embryotoxicity Test. *Toxicol Vitro* **2021**, *72*, 105088. https://doi.org/10.1016/j.tiv.2021.105088.

(2) Ali, S.; Aalders, J.; Richardson, M. K. Teratological Effects of a Panel of Sixty Water-Soluble Toxicants on Zebrafish Development. *Zebrafish* **2014**, *11* (2), 129–141. https://doi.org/10.1089/zeb.2013.0901.

(3) Brotzmann, K.; Escher, S. E.; Walker, P.; Braunbeck, T. Potential of the Zebrafish (Danio Rerio) Embryo Test to Discriminate between Chemicals of Similar Molecular Structure-a Study with Valproic Acid and 14 of Its Analogues. *Arch Toxicol* **2022**, *96* (11), 3033–3051. https://doi.org/10.1007/s00204-022-03340-z.

(4) Brotzmann, K.; Wolterbeek, A.; Kroese, D.; Braunbeck, T. Neurotoxic Effects in Zebrafish Embryos by Valproic Acid and Nine of Its Analogues: The Fish-Mouse Connection? *Arch Toxicol* **2021**, *95* (2), 641–657. https://doi.org/10.1007/s00204-020-02928-7.

(5) Burns, F. R.; Peterson, R. E.; Heideman, W. Dioxin Disrupts Cranial Cartilage and Dermal Bone Development in Zebrafish Larvae. *Aquat Toxicol* **2015**, *164*, 52–60. https://doi.org/10.1016/j.aquatox.2015.04.005.

(6) Chimote, G.; Sreenivasan, J.; Pawar, N.; Subramanian, J.; Sivaramakrishnan, H.; Sharma, S. Comparison of Effects of Anti-Angiogenic Agents in the Zebrafish Efficacy-Toxicity Model for Translational Anti-Angiogenic Drug Discovery. *Drug Devel Ther* **2014**, *8*, 1107–1123. https://doi.org/10.2147/DDDT.S55621.

(7) Chiu, J. Z. S.; Hold, I.; Newman, T. A. C.; Horsfield, J. A.; McDowell, A. Chlorogenic Acid Supplementation Benefits Zebrafish Embryos Exposed to Auranofin. *Pharmaceutics* **2020**, *12* (12). https://doi.org/10.3390/pharmaceutics12121199.

(8) Cunha, V.; Vogs, C.; Le Bihanic, F.; Dreij, K. Mixture Effects of Oxygenated PAHs and Benzo[a]Pyrene on Cardiovascular Development and Function in Zebrafish Embryos. *Env. Int* **2020**, *143*, 105913. https://doi.org/10.1016/j.envint.2020.105913.

(9) Dach, K.; Yaghoobi, B.; Schmuck, M. R.; Carty, D. R.; Morales, K. M.; Lein, P. J. Teratological and Behavioral Screening of the National Toxicology Program 91-Compound Library in Zebrafish (Danio Rerio). *Toxicol Sci* **2019**, *167* (1), 77–91. https://doi.org/10.1093/toxsci/kfy266.

(10) Ellis-Hutchings, R. G.; Settivari, R. S.; McCoy, A. T.; Kleinstreuer, N.; Franzosa, J.; Knudsen, T. B.; Carney, E. W. Embryonic Vascular Disruption Adverse Outcomes: Linking High Throughput Signaling Signatures with Functional Consequences. *Reprod Toxicol* **2017**, *70*, 82–96. https://doi.org/10.1016/j.reprotox.2017.05.005.

(11) Everson, J. L.; Batchu, R.; Eberhart, J. K. Multifactorial Genetic and Environmental Hedgehog Pathway Disruption Sensitizes Embryos to Alcohol-Induced Craniofacial Defects. *Alcohol Clin Exp Res* **2020**, *44* (10), 1988–1996. https://doi.org/10.1111/acer.14427.

(12) Fetter, E.; Baldauf, L.; Da Fonte, D. F.; Ortmann, J.; Scholz, S. Comparative Analysis of Goitrogenic Effects of Phenylthiourea and Methimazole in Zebrafish Embryos. *Reprod Toxicol* **2015**, *57*, 10–20. https://doi.org/10.1016/j.reprotox.2015.04.012.

(13) Fukushima, H. C. S.; Bailone, R. L.; Correa, T.; Janke, H.; De Aguiar, L. K.; Setti, P. G.; Borra, R. C. Zebrafish Toxicological Screening Could Aid Leishmaniosis Drug Discovery. *Lab Anim Res* **2021**, *37* (1), 27. https://doi.org/10.1186/s42826-021-00104-1.

(14) Jarque, S.; Rubio-Brotons, M.; Ibarra, J.; Ordonez, V.; Dyballa, S.; Minana, R.; Terriente, J. Morphometric Analysis of Developing Zebrafish Embryos Allows Predicting Teratogenicity Modes of Action in Higher Vertebrates. *Reprod Toxicol* **2020**, *96*, 337–348. https://doi.org/10.1016/j.reprotox.2020.08.004.

(15) Kalasekar, S. M.; Zacharia, E.; Kessler, N.; Ducharme, N. A.; Gustafsson, J. A.; Kakadiaris, I. A.; Bondesson, M. Identification of Environmental Chemicals That Induce Yolk

Malabsorption in Zebrafish Using Automated Image Segmentation. *Reprod Toxicol* **2015**, *55*, 20–29. https://doi.org/10.1016/j.reprotox.2014.10.022.
(16) Lanham, K. A.; Plavicki, J.; Peterson, R. E.; Heideman, W. Cardiac Myocyte-Specific AHR Activation Phenocopies TCDD-Induced Toxicity in Zebrafish. *Toxicol Sci* **2014**, *141* (1), 141–154. https://doi.org/10.1093/toxsci/kfu111.
(17) Mori, T.; Ito, T.; Liu, S.; Ando, H.; Sakamoto, S.; Yamaguchi, Y.; Tokunaga, E.; Shibata, N.; Handa, H.; Hakoshima, T. Structural Basis of Thalidomide Enantiomer Binding to Cereblon. *Sci Rep* **2018**, *8* (1), 1294. https://doi.org/10.1038/s41598-018-19202-7.
(18) Nawaji, T.; Mizoguchi, N.; Ono, M.; Matuura, T.; Seki, M.; Teraoka, H. Comparing Time-Series of Chemical Concentrations in Zebrafish (Danio Rerio) Embryos/Larvae Exposed to Teratogens with Different Hydrophobicity; Caffeine, Sodium Valproate, and Diethylstilbestrol. *J Toxicol Sci* **2018**, *43* (4), 267–273. https://doi.org/10.2131/jts.43.267.
(19) Nilen, G.; Obamwonyi, O. S.; Liem-Nguyen, V.; Engwall, M.; Larsson, M.; Keiter, S. H. Observed and Predicted Embryotoxic and Teratogenic Effects of Organic and Inorganic Environmental Pollutants and Their Mixtures in Zebrafish (Danio Rerio). *Aquat Toxicol* **2022**, *248*, 106175. https://doi.org/10.1016/j.aquatox.2022.106175.
(20) Simmons, A. E.; Karimi, I.; Talwar, M.; Simmons, T. W. Effects of Nitrite on Development of Embryos and Early Larval Stages of the Zebrafish (Danio Rerio). *Zebrafish* **2012**, *9* (4), 200–206. https://doi.org/10.1089/zeb.2012.0746.
(21) Sogbanmu, T. O.; Nagy, E.; Phillips, D. H.; Arlt, V. M.; Otitoloju, A. A.; Bury, N. R. Lagos Lagoon Sediment Organic Extracts and Polycyclic Aromatic Hydrocarbons Induce Embryotoxic, Teratogenic and Genotoxic Effects in Danio Rerio (Zebrafish) Embryos. *Env. Sci Pollut Res Int* **2016**, *23* (14), 14489–14501. https://doi.org/10.1007/s11356-016-6490-y.
(22) Sornat, R.; Kalka, J.; Faron, J.; Napora-Rutkowska, M.; Krakowian, D.; Drzewiecka, A. Developing a Screening Test for Toxicity Studies of Prenatal Development with the Use of Hydra Attenuata and Embryos of Zebrafish. *Toxicol Rep* **2021**, *8*, 1742–1753. https://doi.org/10.1016/j.toxrep.2021.09.006.
(23) Souder, J. P.; Gorelick, D. A. Ahr2, But Not Ahr1a or Ahr1b, Is Required for Craniofacial and Fin Development and TCDD-Dependent Cardiotoxicity in Zebrafish. *Toxicol Sci* **2019**, *170* (1), 25–44. https://doi.org/10.1093/toxsci/kfz075.
(24) Swiatkowski, W.; Budzynska, B.; Maciag, M.; Swiatkowska, A.; Tylzanowski, P.; Rahnama-Hezavah, M.; Stachurski, P.; Chalas, R. Nicotine and Cytisine Embryotoxicity in the Experimental Zebrafish Model. *Int J Mol Sci* **2023**, *24* (15). https://doi.org/10.3390/ijms241512094.
(25) Teixido, E.; Kiessling, T. R.; Krupp, E.; Quevedo, C.; Muriana, A.; Scholz, S. Automated Morphological Feature Assessment for Zebrafish Embryo Developmental Toxicity Screens. *Toxicol Sci* **2019**, *167* (2), 438–449. https://doi.org/10.1093/toxsci/kfy250.
(26) Walter, K. M.; Miller, G. W.; Chen, X.; Harvey, D. J.; Puschner, B.; Lein, P. J. Changes in Thyroid Hormone Activity Disrupt Photomotor Behavior of Larval Zebrafish. *Neurotoxicology* **2019**, *74*, 47–57. https://doi.org/10.1016/j.neuro.2019.05.008.
(27) Yoo, M. H.; Rah, Y. C.; Park, S.; Koun, S.; Im, G. J.; Chae, S. W.; Jung, H. H.; Choi, J. Impact of Nicotine Exposure on Hair Cell Toxicity and Embryotoxicity During Zebrafish Development. *Clin Exp Otorhinolaryngol* **2018**, *11* (2), 109–117. https://doi.org/10.21053/ceo.2017.00857.
(28) Zamora, L. Y.; Lu, Z. Alcohol-Induced Morphological Deficits in the Development of Octavolateral Organs of the Zebrafish (Danio Rerio). *Zebrafish* **2013**, *10* (1), 52–61. https://doi.org/10.1089/zeb.2012.0830.
(29) Zhang, G.; Marvel, S.; Truong, L.; Tanguay, R. L.; Reif, D. M. Aggregate Entropy Scoring for Quantifying Activity across Endpoints with Irregular Correlation Structure. *Reprod Toxicol* **2016**, *62*, 92–99. https://doi.org/10.1016/j.reprotox.2016.04.012.
(30) Zoupa, M.; Zwart, E. P.; Gremmer, E. R.; Nugraha, A.; Compeer, S.; Slob, W.; van der Ven, L. T. M. Dose Addition in Chemical Mixtures Inducing Craniofacial Malformations in Zebrafish

(Danio Rerio) Embryos. *Food Chem Toxicol* **2020**, *137*, 111117. https://doi.org/10.1016/j.fct.2020.111117.
